# Conservatism in Gun-Shot Wounds of the Abdomen

**Published:** 1896-11

**Authors:** J. M. Fry

**Affiliations:** Wills Point, Texas


For the Texas Medical Journal.

### CONSERVATISM IN GUN-SHOT WOUNDS OF THE ABDOMEN.

BY J. M. FRY, M. D., WILLS POINT, TEXAS.

[Read before the Wills Point Medical Society, October 12, 1896.]

FOR some time the glamour of success accomplished by means of modern aseptic surgery has had a tendency to make operators more radicial in their views, and bolder in their methods.

It has been my conviction that some steps should be taken in the direction of greater conservatism in abdominal surgery.

The current of opinion has now, however, set in the other way, and the pendulum of opinion is swinging back to a more rational position.

Prof. N. Senn has lately said:

"The present tendency is to adopt more and more conservative measures in place of mutilating operations. It is no longer the man who can amputate a limb successfully, and obtain satisfactory results, that is entitled to distinction, but it is the humbler worker in the science of surgery that interprets these pathological conditions correctly, and subjects his patients to non-mutilating, conservative measures.

"The successful surgeon is neither the one who cuts indiscriminately, nor he who fears to cut at all, but he who judiciously selects his cases for operation."

Valentine Mott once said: "Be bold, but cautious."

Cautious in the selection of cases for operation, cautious in every detail; bold to act in emergency, or when action is indicated.

And while I insist that no operation be attempted unless the indications positively demand it; it will do the patient nor surgeon no benefit to tempòrize with cases that require an operation.

Frederick Lippincott, M. D., of Philadelphia, in his inaugural prize thesis on the subject of gun-shot wounds of the abdomen, draws the following conclusions:

"1. In about ninety per cent of the penetrating wounds of the abdomen the viscera are involved.

"2. In the majority of cases of abdominal gun-shot wounds the intestinal lesions are multiple.

"3. When the viscera are wounded, fæces and gas or finding the ball in the evacuations are the only pathognomonic symptoms.

"4. Nature is capable of effecting complete repair in wounds of the viscera by prolapse of mucus membrane, exudation of plastic lymph, bringing a healthy surface over the rent in the gut, and finally cicatrization.

"5. Statistics do not show a better mortality by the operative than they do by the conservative treatment.

"6. Wounds of the stomach and small intestines are more grave than those of the large intestines.

"7. Shock of itself is not a symptom of internal hemorrhage.

"8. The most common cause of death in gun-shot wounds of the abdomen is septic peritonitis, although deaths from shock and hemorrhage are not rare.

"9. The use of hydrogen gas by rectum as a means of diagnosis is not infallible.

"10. Satistics do not justify abdominal section except in well selected cases, and particularly when there is pronounced hemorrhage.

"11. So far laporatomy has increased the mortality of gunshot wounds of the abdomen.

"12. It is necessary for localized peritonitis to occur in order to attach the wounded gut to some healthy peritoneal surface.

"13. The indications for treatment are, to promote reaction, control hemorrhage by pressure, or abdominal section, stimulate hypordermically, nothing by mouth, feed by rectum until adhesions have perfectly formed."

I consider it paramount to determine positively and early if an operation is demanded.

There are, in my judgment, but two conditions in gun-shot wounds of the abdomen that justify surgical interference.

1st. Dangerous internal hemorrhage.

2nd. Wounds of the stomach or intestines large enough to admit of extravasation.

Intra-abdominal pressure is simply muscular tension. In gun-shot wounds the bowel contents do not extravasate rapidly. The reason is that the pressure in the gut lumen and peritoneal cavity is the same.

The following quotations from the American Text Book on Surgery serves to show how difficult it is to decide just what cases require operation:

"It must, however, be admitted, that in the absence of serious visceral lesions, penetrating wounds of the abdomen are injuries from which the patients are very likely to recover without operative treatment, and that when such patients are subject to laparotomy, death may occur solely in consequence of the operation. * * *

"The general symptoms in cases of penetrating punctured wounds of the abdomen, with the exception of those due to profuse hemorrhage, furnish absolutely no reliable information in the differentiation between simple penetrating wounds and penetrating wounds compicated by visceral injuries. * * *

"With the exception of the last-mentioned symptom (the escape of the contents of the stomach or intestines through the external wound) and the indication pointing to the necessity of arresting internal hemorrhage, there is nothing about the local or general symptoms in cases of penetrating wounds of the abdomen that enable the surgeon to decide with any degree of positiveness whether visceral injuries exist or not, and consequently, whether laparotomy should or should not be performed. * * *

"Severe shock may attend even a non-penetrating wound, and it may be absent, or at least not well marked, in cases of multiple perforation of the intestines. * * *

"Vomiting takes place as frequently in wounds of the abdominal parieties and in simple penetrating wounds as when the viscera has been penetrated. * * *

"The pulse is slow and compressible in all cases, and nothing characteristic in its qualities is observed even if the stomach or intestines have been injured. * * *

"Pallor is present in all penetrating wounds of the abdomen soon after the receipt of the injury, and it is only more pronounced when produced, at least in part, by sudden and severe internal hemorrhage. * * *

"Pain is a very unreliable and often misleading symptom, as it may be moderate or almost completely absent soon after the injury has been inflicted, even when multiple perforations are present." * * *

External hemorrhage usually comes from the parietes, and when profuse might only indicate that a more or less important vessel has been cut.

Some surgeons place great stress on the absence of liver dullness as an evidence of visceral perforation, but if the external wound is large gas will leak through without giving any evidence of perforation. This is an important subject from a medico-legal, as well as a surgical, standpoint.

